# Does Air Pollution Affect Prosocial Behaviour?

**DOI:** 10.3389/fpsyg.2022.752096

**Published:** 2022-03-28

**Authors:** Sheng Zeng, Lin Wu, Zenghua Guo

**Affiliations:** ^1^School of Sociology, Wuhan University, Wuhan, China; ^2^School of Marxism, Hubei University of Economics, Wuhan, China

**Keywords:** air pollution, prosocial behaviour, individual blood donation, component blood donation, whole blood donation

## Abstract

Air pollution has become a serious issue that affects billions of people worldwide. The relationship between air pollution and social behaviour has become one of the most widely discussed topics in the academic community. While the link between air pollution and risk-averse and unethical behaviours has been explored extensively, the relationship between air pollution and prosocial behaviour has been examined less thoroughly. Individual blood donation is a typical form of prosocial behaviour. We examined the effect of air pollution on prosocial behaviour using the Poisson regression quasi-maximum likelihood (PQML) based on the panel data related to air pollution and blood donations. We also employed a set of control variables and robustness checks. The findings indicate that air pollution does not affect whole blood donation, although it does affect component blood donation. We also find that the effect of air pollution on blood donation is heterogeneous in terms of gender, age, and other factors. These results show that the relationship between air pollution and prosocial behaviour is limited. Not all types of prosocial behaviour are affected by air pollution, perhaps because air pollution affects only specific psychological motivations and because different types of prosocial behaviour have different motivations.

## Introduction

Air pollution is a serious issue that affects billions of people worldwide. In 2019, David Boyd, the UN Special Rapporteur on Human Rights and the Environment, stated that the lives and health of more than 6 billion people around the world are seriously threatened by chronic exposure to highly polluted air, and as many as 7 million people die each year from indoor and outdoor air pollution. The conclusion that air pollution negatively impacts physical health has been supported by researchers in many disciplines and fields (McGlade and Landrigan, 2019; Qiu et al., 2019; Chen and Chen, 2020). Long-term exposure to air pollution increases the risk of premature death (Lelieveld et al., 2015), cardiovascular and cerebrovascular diseases (Lim et al., 2019; Mannucci et al., 2019), hypertension (Avila-Palencia et al., 2019; Curto et al., 2019; Li et al., 2020), respiratory disease (Losacco and Perillo, 2018; Tang et al., 2018), and chronic disease (Jiang et al., 2016; Hooper et al., 2018). Air pollution may also contribute to feelings of depression and anxiety (Gu et al., 2019; Fan et al., 2020; Lu et al., 2020). In addition, exposure to air pollution can harm people’s cognitive functioning, which includes attention, visuoconstruction, memory, math ability, reading comprehension, verbal intelligence, and non-verbal intelligence (Zhang et al., 2018; Adar et al., 2019). The impact of air pollution on cognitive functioning encompasses the entire life span, from prenatal development, childhood, and youth to young and old adulthood (Sun and Gu, 2008; Perera et al., 2009; Mohai et al., 2011; Lertxundi et al., 2015; Yorifuji et al., 2016). In addition, most people tend to engage in risk-averse behaviours to prevent the impact of air pollution. When air pollution is high, people tend to avoid outdoor activities, such as cycling, trips, and park usage (Bresnahan et al., 1997; Neidell, 2009; Moretti and Neidell, 2011; Saberian et al., 2017). Individuals in polluted regions also show increased interest in emigration and defensive expenditure. Qin and Zhu (2018) found that a 100-point increase in the air quality index (AQI) predicted a 2.3–4.8% increase in internet searches for “emigration” the next day. Zhang and Mu (2018) found that, in China, a 100-point increase in AQI increased the consumption of all masks by 54.5% and anti-PM_2.5_ masks by 70.6%. What is even more concerning is that air pollution is associated with increased aggressive and unethical behaviours (Herrnstadt et al., 2016). For example, using 10-year panel data on counties in the contiguous United States, Burkhardt et al., 2019a found that an increase in PM_2.5_ increased violent crime rates, especially assaults. Lu et al. (2018) found that air pollution predicted both violent crimes (murder, rape, robbery, and assault) and property crimes (burglary and motor vehicle theft). Bondy et al. (2020) reached similar conclusions using data from London.

The effect of air pollution is widespread and is not limited to the domains covered by the studies reviewed in this article (Lu, 2020). For example, some scholars have found that air pollution decreases the labour supply by increasing absenteeism (Hanna and Oliva, 2015). The impact of air pollution on human society is far-reaching, especially on social behaviour. Hence, the potential role of air pollution cannot be ignored when exploring a given social behaviour. Scholars from various disciplines and fields agree that air pollution induces risk-averse and unethical behaviours. However, researchers have neglected the relationship between air pollution and prosocial behaviour. Research exploring the relationship between air pollution and prosocial behaviour is relatively scarce. Prosocial behaviours are voluntary and benefit other people, such as charitable donations, justice, cooperation, trust, and altruistic punishment (Luo, 2018). Prosocial behaviour plays an important role in both individuals and society. For individuals, prosocial behaviour enhances their self-moral identity, social self-efficacy, and value of social commitment (Patrick et al., 2018). For society, prosocial behaviour is a powerful means of promoting productivity, increasing social wealth, and resisting social risk (Thielmann et al., 2020). Numerous systematic studies have examined prosocial behaviour. However, few scholars have directly studied the relationship between air pollution and prosocial behaviour. Exploring the association between air pollution and prosocial behaviour using a quasi-experimental approach, Zhao et al. (2020) found that the more polluted the air, the less likely Chinese university students were to engage in prosocial behaviour. Their finding implies that air pollution is significantly negatively associated with prosocial behaviour. However, their research has not provided a complete picture of the complex association between air pollution and prosocial behaviour. On the one hand, the quasi-experimental study allowed us to better observe the effects of air pollution on honest behaviour in the nearest actual situation. However, there are still differences from a completely objective and realistic environment. Obviously, there is a corresponding bias in the study findings. On the other hand, prosocial behaviour is so complex that it is impossible to determine its association with pollution based on only a few studies. Thus, examining the association between air pollution and prosocial behaviour is essential. In addition, Zhao et al. (2020) mainly use Chinese university students as the research participants. It remains to be tested whether the research findings could be extended to society as a whole. In this article, we explore the relationship between air pollution and prosocial behaviour to fill these research gaps.

Our study has important implications for research. First, the previous studies have explored the relationship between air pollution and antisocial or unethical behaviour. Comparatively, the relationship between air pollution and prosocial behaviour has not been thoroughly explored. Therefore, our analysis of the relationship between air pollution and prosocial behaviour could fill a research gap in this field. Second, most prior studies measure prosocial behaviour using questionnaires, experimental methods, or quasi-experimental methods. These methods constitute the dominant paradigm in the current research and are very effective. However, these studies all had methodological biases. It is extremely easy for researchers to incorporate their value judgements or social expectations into the study. This often results in self-reporting or social desirability biases when prosocial behaviour is reported or observed in a context that may rouse such biases in participants. Our study considers actual air pollution and prosocial behaviour occurring in a real environment, which effectively compensates for the biases in the previous studies. Third, we use blood donations occurring in natural scenarios to represent prosocial behaviour, which reflects the complex association between air pollution and prosocial behaviour more accurately than what is attempted in prior studies and thus deepens our understanding of that relationship.

The remainder of this article is organised as follows. The next section outlines the study’s data and provides the summary statistics. Section “Model Specification” presents our econometric model. Section “Results” presents the results of the study. Finally, Section “Discussion” discusses the results and provides conclusions.

## Data

### Data Collection

Our final dataset spanning 13 administrative districts was merged with data on individual blood donation, air pollution, and weather in H city in 2018. We used a unique method to identify the relationship between air pollution and prosocial behaviour. As mentioned, the questionnaire, experimental, and quasi-experimental methods all involve a certain degree of methodological bias. To avoid this shortcoming, this study used individual blood donations that occur in real-life every day as a predictive indicator of prosocial behaviour. Blood donation is the act of volunteering to give blood for the public good to save lives without receiving compensation for necessary costs such as transportation and lost work or any other remuneration. In China, blood donation is an act of selflessness and dedication to saving lives. In other words, blood donation is a typical prosocial behaviour that cannot be traded. Using individual blood donations to measure prosocial behaviour is more objective and realistic than the previous methods. Several researchers have found that the effects of air pollution on physical, mental, and social behaviours are closely related to particulate matter (PM) in the air (Schlenker and Walker, 2016; Di et al., 2017; Archsmith et al., 2018; Deryugina et al., 2019; Nephew et al., 2020). PM is a mixture of many different organic and inorganic components (Naeher et al., 2007; Craig et al., 2012; Austin et al., 2013). The studies have found that the specific components of PM can lead to systematic inflammation associated with adverse health outcomes (Brook et al., 2010; Zahran et al., 2017) and unethical behaviours (Burkhardt et al., 2019a). Burkhardt et al. (2019a) found that a 1 μg/m^3^ increase in the monthly average PM_2.5_ increases monthly violent crime rates by 0.53%. The harmful effects of air pollution on physical health, mental health, cognitive function, and social behaviour are largely due to PM_2.5_. Some scholars have used PM_2.5_ as a predictive indicator of air pollution, such as Berman et al. (2019) and Burkhardt et al. (2019a). Based on the approach of these studies, we use PM_2.5_ as a predictive indicator of air pollution.

We examined the relationship between air pollution and prosocial behaviour by merging three datasets. First, we collected data on individual blood donations in 2018 from the blood centre. The dataset records basic information about individual blood donations at the district-day level, which includes donor’s gender, age, category, type, time, and specific location of the blood donation. Group blood donation is a kind of blood donation programme that is organised by units and social groups that can heavily influence this donation. This results in potential self-report or social desirability biases when prosocial behaviour is either reported or observed in a context. This type of donation does not occur randomly based on the participants’ wishes, unlike individual blood donations. Thus, this study mainly uses individual blood donations as a predictive indicator of prosocial behaviour. Individual blood donations comprise whole blood and component blood donations. Based on the classification of individual blood donation, we computed the count of blood donations according to gender, age, type of blood donation, and other characteristics. We ultimately obtained a dataset of individual blood donations sorted by administrative district and date. Table 1 presents several concepts related to the individual blood donations.

**TABLE 1:** Categories of blood donation.

Category	Brief introduction
Whole blood donation	The mixture formed by collecting human blood into the blood collection bag, which includes all the components of blood cells and plasma.
Component blood donation	Healthy citizens donate a certain component of blood through a blood separator. The donated components can be platelets, granulocytes, or peripheral blood stem cells. The most common form in China is the donation of platelets.
Fist-time blood donor	Blood donors who are donating for the first time.
Regular blood donor	Blood donors who have donated 3 times and at least once in the past year.
Repeat blood donor	Blood donors who have donated twice and at least once in the past 2 years, or who have donated 3 times or more, and donated the last time within a year or less than 2 years ago.
Returning blood donor	Blood donors who have donated in the past, but have not donated for 2 years or more before donating blood this month.

Second, we collected data on the PM_2.5_ pollutant index from the Bureau of Ecology and Environment. The pollution index was converted according to the PM_2.5_ concentration monitored daily. The Bureau of Ecology and Environment has published data on the PM_2.5_ pollutant index on its website since November 2015. A higher PM_2.5_ pollutant index means a higher PM_2.5_ concentration and thus greater air pollution. Due to the maintenance issues with some of the monitors, data for a few days may be missing. However, the number of missing data is very small, and it is not sufficient to affect the representativeness of the data. We matched the data based on the corresponding relationship between air pollution monitoring points and administrative districts and sorted the daily PM_2.5_ pollutant index of the administrative districts.

Third, we collected weather data using a historical Chinese meteorological website. This website provides data on China’s ground weather since 1942 for free. We set wind direction and weather phenomena as dummy variables. The wind direction in our dataset contained seven specific wind directions: South, North, Southeast, Southwest, Northwest, Northeast and East winds. Thus, we divided wind direction into seven dummy variables. South, North, Southeast, Southwest, Northwest, Northeast or East winds were coded as 1; no wind was coded as 0. For the weather phenomenon variables, we coded sunny and cloudy days (i.e., good weather) as 0 and coded overcast, foggy, light rain, moderate rain, heavy rain, sleet, and moderate snow (i.e., bad weather) as 1.

### Summary Statistics

Table 2 displays the summary statistics for several key variables used in our research. As can be seen from the data we collected from the blood centre, 1,61,655 individual blood donations were made in 2018 according to the administrative districts. The number of blood donations per day varies considerably from district to district because the number of blood donation sites also varies across administrative districts. We collected 338 observation cases that included missing air pollution data from the Bureau of Ecology and Environment. We excluded these cases from the final dataset to ensure that the data were as objective and accurate as possible. As a result, a total of 4,407 observations and 1,46,145 individual blood donations were included in the final dataset after the missing data were excluded.

**TABLE 2:** Summary statistics of some key variables.

Variable	Mean	Std. dev.	Min	Max	N
Blood donation	33.16	49.23	0	366	4,407
Whole blood donation	21.54	33.61	0	304	4,407
Male whole blood donation	4.791	5.710	0	99	4,407
Female whole blood donation	2.944	3.844	0	57	4,407
First whole blood donation	4.331	6.036	0	105	4,407
Regular whole blood donation	1.964	2.245	0	20	4,407
Returning whole blood donation	0.462	0.733	0	7.667	4,407
Repeat whole blood donation	0.962	1.438	0	23	4,407
Component blood donation	11.63	30.08	0	233	4,407
Male component blood donation	8.242	21.82	0	171	4,407
Female component blood donation	2.185	6.068	0	62	4,407
First component blood donation	0.317	1.218	0	33	4,407
Regular component blood donation	9.444	25.20	0	227	4,407
Returning component blood donation	0.027	0.179	0	3	4,407
Repeat component blood donation	0.639	2.062	0	24	4,407
PM_2.5_ pollutant index	59.85	33.56	5	303	4,407
Weather phenomena	0.468	0.499	0	1	4,407
Daily max temperature (C)	22.44	10.24	0	39	4,407
Daily min temperature (C)	13.78	8.957	−3	30	4,407
**Wind direction**					
South wind	0.055	0.229	0	1	4,407
North wind	0.066	0.248	0	1	4,407
Southeast wind	0.179	0.383	0	1	4,407
Southwest wind	0.147	0.354	0	1	4,407
Northwest wind	0.222	0.416	0	1	4,407
Northeast wind	0.331	0.470	0	1	4,407
East wind	0.001	0.034	0	1	4,407
Wind rating	2.160	0.945	1	7	4,407

*Blood donation reported as counts per day. Weather phenomena variable is a dummy variable; therefore, the means indicate that, for example, 46.8% of the observations in the dataset are bad weather using the raw weather phenomena variable. Wind direction variables are also dummy variables, and their interpretation is similar to that of the weather variable.*

In our final dataset, individual blood donations were reported in accordance with the actual number of blood donations per day in each administrative district. As shown in Table 2, the average daily number of individual blood donations was 33.16 at the district level, among which the highest number of individual blood donations in a single day was 336. Similarly, the average daily number of whole blood donations was 21.54 at the district level, among which the highest number of whole blood donations per day was 304. The average daily number of component blood donations was 11.63 at the district level, among which the highest number of component blood donations in a single day was 233. In addition to displaying the summary statistics for whole blood donations and component blood donations, we also report descriptive statistics for sex, weather, and blood donation type. For example, the average daily number of men in the blood donation component at the district level was 8.242, among which the highest number of men in the blood donation component in a single day was 171. The average daily number of regular donors for component blood donation at the district level was 9.44, among which the highest number of regular blood donors for this donation type in a single day was 227. Table 2 also shows that the daily average PM_2.5_ pollutant index was 59.85 at the district level, the lowest PM_2.5_ pollutant index was 5, and the highest was 303 in a single day. In addition, the weather data reported in Table 2 show that the average daily maximum temperature and daily minimum temperature were 22.44 and 13.78^°^C, respectively, at the district level. The average daily wind rating at the district level was 2.16°; the lowest and highest wind ratings on a single day were 1° and 7°, respectively. As we have explained the weather phenomena and wind direction variables in the notes in Table 2, we will not go into further detail here.

## Model Specification

Our baseline econometric specification examined the relationship between air pollution and prosocial behaviour, in which prosocial behaviour was measured as individual blood donation, as follows:


(1) $$Blood_{dt}^{j}=\gamma_{PM}^{j}PM25_{dt}+X_{\;dt}{\text{β}}^{j}+\lambda_{d}+\xi_{t}+\in_{dt}^{j}$$

The dependent variable $Blood{}_{d\,t}^{j}$ is the count of blood donation type *j* in district *d* on day *t*, and *PM*25_*dt*_ is the corresponding PM_2.5_ pollutant index in district *d* on day *t*. *X*_*dt*_ is the covariate *X* in district *d* on day *t*. *X* contains a set of covariates that may affect the estimation results, such as daily maximum temperature, daily minimum temperature, weather, wind direction, and wind rating. Previous studies have found a strong correlation between the weather and air pollution (Burkhardt et al., 2019b). In addition, weather conditions are closely related to psychological activity and social behaviour (Zhao et al., 2020). Therefore, we also included a set of weather-related data as covariates in our model. λ_*d*_ denotes the district fixed effect. ξ_*t*_ denotes fixed effects for the month and day of the week. *∈^j*_*dt*_ is the error term of blood donation type *j* in district *d* on day *t*. As individual blood donations are non-negative count variables with overdispersion, we employed the Poisson regression quasi-maximum likelihood (PQML) estimation, which produces consistent parameter estimates, assuming that the conditional mean function is correctly specified, even when the dependent variable is overdispersed (see Wooldridge (2010), Section 18.2 for details and Moeltner et al. (2013) for an example of implementation). To ensure correct inferences, standard errors (SEs) were clustered at the district level. In addition, the Akaike information criterion (AIC) and the Bayesian information criterion (BIC) are not meaningfully different across models. When the PQML standard errors are not corrected for overdispersion, the standard errors are smaller as would be expected if overdispersion is present (Burkhardt et al., 2019b). Given this result, our preferred specification is the fully robust PQML model with standard errors clustered at the district level. This model specification was similar to that of Burkhardt et al. (2019b). As the number of blood donation stations varies from district to district, the number of blood donations used in Equation (1) is the relative number of donations.

Air pollution is likely endogenous in Equation (1) because there may be unobservable factors between PM_2.5_ and individual blood donation that vary between districts and times. For example, PM_2.5_ and individual blood donation may be correlated with population density, population structure, industrial activity, traffic density, household consumption, etc. Failure to control for these observations effectively may lead to a biased estimation of γ_*PM*_. By including district-fixed effects, the model specification in this study can compare the number of different blood donations between days with different PM_2.5_, as the potential for biased estimation is eliminated. We also included a month fixed effect to control for seasonal variation in PM_2.5_ and the number of blood donations. In addition, there may be periodic co-movement between PM_2.5_ and the number of blood donations; these are not fully identified by the month fixed effects, which are constant across districts. For example, we would like to know the difference between busy weekdays and quiet Sundays in terms of both PM_2.5_ and the number of blood donations. To identify such time-varying variables, dummy variables for the day of the week were included in Equation (1) to solve the potential short-run seasonality problem described above.

We also controlled for weather variables, such as daily maximum temperature and daily minimum temperature, and used dummies for weather phenomena such as wind direction and rating. A body of research has shown that there is a close relationship between weather conditions, air pollution, and unethical behaviour (Zannetti, 2013; Bondy et al., 2020). Although our focus is on prosocial behaviour rather than unethical behaviour, blood donation is also a type of social behaviour. Thus, we have a reason to expect a connection between weather and individual blood donation, making it necessary to control for weather variables.

## Results

In this section, we first discuss the link between air pollution and prosocial behaviour, measured as PM_2.5_, and individual blood donation. Next, we examine the strength and consistency of the main results using several specifications. Then, we explore the heterogeneity of the relationship between air pollution and prosocial behaviour by disaggregating blood donation in terms of gender and age and exploring the moderating role of temperature. Finally, we conduct robustness checks to examine the consistency and reliability of the results.

### Main Results

Table 3 presents the analysis results on the association between air pollution and prosocial behaviour.

**TABLE 3:** Main results of Poisson regression.

	(1)	(2)	(3)
	Unpaid	Whole	Component
	blood donation	blood donation	blood donation
PM_2.5_	−0.0001	0.0007	−0.0012***
	(0.0005)	(0.0007)	(0.0001)
Covariates	Y	Y	Y
District FE	Y	Y	Y
Month FE	Y	Y	Y
Dow FE	Y	Y	Y
N	4,407	4,407	644

All regressions include control variables, such as daily maximum temperature, daily minimum temperature, weather, wind directions, wind rating and holiday. SEs are clustered at the district level. “Y” indicates that these variables are included as predictors in the model. *** denotes significance at the 0.1% levels.

**FIGURE 1:**
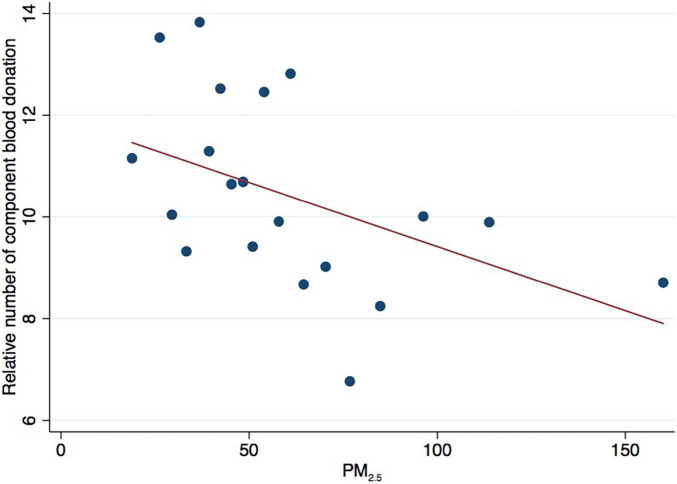
Binned scatter relationship between PM_2.5_ and relative number of component blood donation.

As shown in Table 3, we did not find a significant relationship between PM_2.5_ and overall individual blood donation (see Column 1 in Table 3). Similarly, we found no significant relationship between PM_2.5_ and whole blood donation (see Column 2 in Table 3). By contrast, we found a statistically significant relationship between PM_2.5_ and component blood donation, which indicates that blood donation was driven entirely by component blood donation. These results indicate that air pollution does not affect all prosocial behaviours but only specific types, such as component blood donation. Figure 1 presents the negative relationship between PM_2.5_ and component blood donation.

Based on the main results presented in Table 3, we explored the relationship between PM_2.5_ and component blood donation. In this section, we examine the strength and consistency of the association between PM_2.5_ and blood donation using several sets of fixed effects (FE). As shown in Table 4, we did not find a statistically significant relationship between PM_2.5_ and component blood donation, as presented in Columns 1 and 2, which included the district fixed effect, day-of-the-week fixed effect, and district-by-month-by-day of the week fixed effect, respectively. However, we found a significant effect in Columns 3 and 4, in which changes in PM_2.5_ affect the number of component blood donations (*t* = 0.0012, *p* < 0.05). The model presented in Column 3 of Table 4 includes the district fixed effect, month fixed effect, and day-of-the-week effect. The model presented in Column 4 of Table 4 includes the district-by-month fixed effect and the day-of-the-week fixed effect. Based on the results presented in Table 4, our preferred specification includes either the set of fixed effects presented in Column 3 or the set of fixed effects presented in Column 4.

**TABLE 4:** Main results of component blood donation.

	(1)	(2)	(3)	(4)
PM_2.5_	−0.0041	0.0000	−0.0012***	−0.0012***
	(0.0060)	(0.0003)	(0.0001)	(0.0001)
Covariates	Y	Y	Y	Y
District FE			Y	
Month FE	Y		Y	
District-month FE				Y
Dow FE	Y		Y	Y
District-month-dow FE		Y		
N	644	632	644	644

The dependent variable is the blood donation component. All regressions include daily maximum temperature, daily minimum temperature, weather, wind direction, wind rating, and holidays. The SEs are clustered at the district level. “Y” indicates that these variables have been included as predictors in the model. *** denotes significance at the 0.1% levels.

### Heterogeneity Check

As shown in Table 3, we found no significant relationship among PM_2.5_, individual blood donations, and whole blood donations. Thus, in this section, we discuss the heterogeneity check results for blood donation. As shown in Table 5, the results presented in Columns 1, 3, and 4 indicate that no relationship was found between changes in PM_2.5_ and the first, returning, and repeat components of blood donation. In addition, we found a significant relationship between PM_2.5_ and regular component blood donation (*t* = −0.0014, *p* < 0.05). The positive association between PM_2.5_ and component blood donation is driven mainly by regular component blood donation.

**TABLE 5:** Heterogeneous relationship in different types of component blood donation.

	(1)	(2)	(3)	(4)
	First	Regular	Returning	Repeat
PM_2.5_	0.0024	−0.0014***	−0.0019	0.0004
	(0.0012)	(0.0001)	(0.0101)	(0.0008)
Covariates	Y	Y	Y	Y
District FE	Y	Y	Y	Y
Month FE	Y	Y	Y	Y
Dow FE	Y	Y	Y	Y
N	644	644	644	644

The dependent variable in column 1 is first component blood donors. The dependent variable in column 2 is regular component blood donors. The dependent variable in column 3 is returning component blood donors. The dependent variable in column 4 is repeat component blood donors. SEs are clustered at the district level. “Y” indicates that these variables are included as predictors in the model. *** denotes significance at the 0.1% levels.

Furthermore, we examined gender differences in the relationship between PM_2.5_ and component blood donation. The results reported in Columns 1 and 2 of Table 6 show that a significantly positive relationship was found between PM_2.5_ and component blood donation in both the male and female cohorts. Importantly, the coefficients shown in Columns 1 and 2 were almost the same, which indicates that no gender difference was observed in the association between PM_2.5_ and component blood donation.

**TABLE 6:** Heterogeneous relationship in different gender.

	(1)	(2)
	Male	Female
PM_2.5_	−0.0012***	−0.0012***
	(0.0001)	(0.0000)
Covariates	Y	Y
District FE	Y	Y
Month FE	Y	Y
Dow FE	Y	Y
N	644	644

In addition, we explored age differences in the relationship between PM_2.5_ and blood donation. As shown in Table 7, we divided age into three groups. Columns 1, 2, and 3 show the results for the 18–29, 30–45, and 46–60-year-old groups, respectively. The results shown in Column 1 indicate that no relationship was found between PM_2.5_ and component blood donation in the 18–29 group. By contrast, the results reported in Columns 2 and 3 indicate that a statistically significant relationship was found between PM_2.5_ and component blood donation in the 30–60 group. As air pollution increased, the number of blood donors declined significantly among those over 30. Since the coefficients of Columns 2 and 3 were almost the same, the results indicate that there was no significant difference in the relationship between PM_2.5_ and component blood donation for the 30–45 and 46–60 groups.

**TABLE 7:** Heterogeneous relationship in different age.

	(1)	(2)	(3)
	Age (18–29)	Age (30–45)	Age (46–60)
PM_2.5_	−0.0000	−0.0022***	−0.0022***
	(0.0001)	(0.0003)	(0.0003)
Covariates	Y	Y	Y
District FE	Y	Y	Y
Month FE	Y	Y	Y
Dow FE	Y	Y	Y
N	644	644	644

The dependent variable in column 1 is component blood donors aged 18–29. The dependent variable in column 2 is component blood donors aged 30–45. The dependent variable in column 3 is component blood donors aged 46–60. SEs are clustered at the district level. “Y” indicates that these variables are included as predictors in the model. *** denotes significance at the 0.1% levels.

In this section, we examine the heterogeneity between PM_2.5_ and component blood donation according to sex and age by interacting gender and age to explore whether the correlation between PM_2.5_ and component blood donation differs across gender groups at different ages. In Table 8, the result in Column 1 indicates that a significantly negative relationship was found between PM_2.5_ and component blood donation among men aged 30–45. Similarly, we found a significant relationship between PM_2.5_ and blood donation among men aged 46–60 (as shown in Column 3). It is worth noting that the coefficients were almost the same among men aged 30–45 and 46–60. In addition, we found a significantly negative relationship between PM_2.5_ and component blood donation among women aged 30–45 and 46–60 (as shown in Columns 2 and 4). The coefficients of these two models were also almost the same, which indicates that there was no difference in the relationship between PM_2.5_ and component blood donation among men and women in the 30–45 and 46–60 groups, respectively. However, differences were observed in the association between PM_2.5_ and blood donation between men and women in the same age group. In general, when PM_2.5_ increased, blood donations declined among both men and women over 30. Only the magnitude of the relationship between PM_2.5_ and blood donation differs between men and women in the same age group.

**TABLE 8:** Interactions with gender and age.

	(1)	(2)	(3)	(4)
	Male 30–45	Female 30–45	Male 46–60	Female 46–60
PM_2.5_	−0.0020***	−0.0022***	−0.0020***	−0.0022***
	(0.0001)	(0.0006)	(0.0006)	(0.0006)
Covariates	Y	Y	Y	Y
District FE	Y	Y	Y	Y
Month FE	Y	Y	Y	Y
Dow FE	Y	Y	Y	Y
N	644	644	644	644

The dependent variable in column 1 is male component blood donors aged 30–45. The dependent variable in column 2 is female component blood donors aged 30–45. The dependent variable in column 3 is male component blood donors aged 46–60. The dependent variable in column 4 is female component blood donors aged 40–60. SEs are clustered at the district level. “Y” indicates that these variables are included as predictors into the model. *** denotes significance at the 0.1% levels.

### Further Results

**FIGURE 2:**
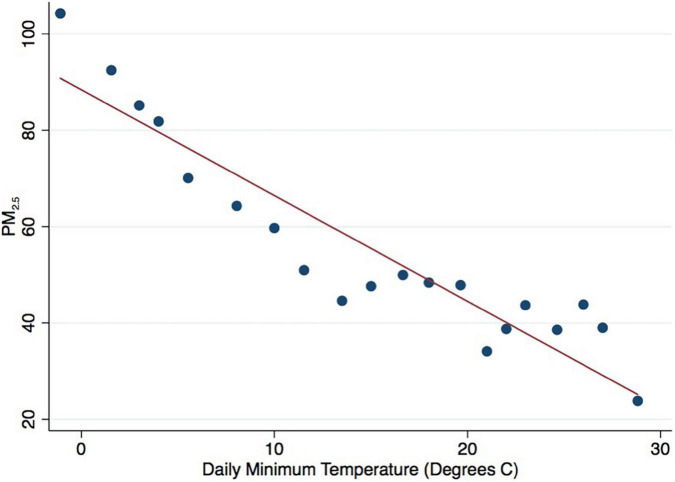
Binned scatter relationship between PM_2.5_ and daily minimum temperature.

A large body of the literature has shown a close link among temperature, air pollution, and social behaviours (Lynott et al., 2017; Van Assche et al., 2017; Zhang et al., 2018; Berman et al., 2019). Thus, we tested the moderating role of temperature in the relationship between air pollution and prosocial behaviour. The results in Table 5 show that PM_2.5_ was closely associated with regular blood donation only for component blood donation. In this section, we test the moderating role of temperature in the relationship between PM_2.5_ and regular component blood donation. We found no significant moderating role of daily maximum temperature in the relationship between PM_2.5_ and regular blood donation and thus do not report the detailed results here. By contrast, we found a significant moderating role of daily minimum temperature in the association between PM_2.5_ and regular blood donation. As shown in Table 9, the daily minimum temperature was divided into three groups. The results indicate a significant relationship between PM_2.5_ and regular blood donation when the daily minimum temperature was in the first and second quartiles. Based on this, we calculated the specific values of the quartiles and found that there was a significant link between PM_2.5_ and regular blood donation when the daily minimum temperature was below 15°C: The lower the daily minimum temperature, the stronger the association between PM_2.5_ and regular blood donation. When the daily minimum temperature exceeded 15°C, the relationship between PM_2.5_ and regular blood donation disappeared. Figure 2 presents the results on the relationship between PM_2.5_ and daily minimum temperature. The results indicate a negative link between PM_2.5_ and daily minimum temperature: the lower the daily minimum temperature, the higher the PM_2.5_.

**TABLE 9:** Moderating role of daily minimum temperature.

	(1)
	**Regular component blood donation**

PM_2.5_* 1 (first min temp quartile)	−0.0031**
	(0.0012)
PM_2.5_* 1 (second min temp quartile)	−0.0005**
	(0.0002)
PM_2.5_* 1 (third min temp quartile)	−0.0016
	(0.0018)
Covariates	Y
District FE	Y
Month FE	Y
Dow FE	Y
N	644

All other controls are included, such as daily minimum temperature, weather, wind direction, wind rating, and holiday. SEs are clustered at the district level. “Y” indicates that these variables are included as predictors in the model. ** denotes significance at the 1% levels. * denotes multiplication operator.

### Robustness Check

We conducted a robustness check using several fixed effects in the previous section (as shown in Columns 3 and 4 in Table 4). The results show that our estimation was relatively robust when we separately controlled for the district fixed effects, month fixed effects, and day-of-the-week fixed effects. In other words, we could obtain more reliable results by including these fixed effects in the model. We included these fixed effects for the following reasons. First, the level of economic development varies from district to district, as do population movement transport conditions. This means that district differences must be considered when performing the model estimation. Thus, we added a district fixed effect to our model. Second, in addition to the district fixed effect, the month fixed effect and the day-of-the-week fixed effect should be included in our model. In H city, blood donations vary on a monthly basis. During the winter and summer holidays, the number of blood donations may be lower than that in other months. Similarly, blood donations differ across days of the week. They may increase on weekends. Thus, we also added a month fixed effect and a day-of-the-week fixed effect. As we collected only 1 year’s worth of data, we do not include a year fixed effect in our model. The results presented in Table 4 indicate how these fixed effects should be included in the model to obtain a reliable estimation.

We thus tested the robustness of our results using different model specifications. Our main model specification was PQML. In this section, we replace our main model specification with the panel Poisson regression fixed effect and negative binomial to re-examine the results. This robustness check strategy was inspired by Burkhardt et al. (2019b). In general, these two models are well suited for our study. As shown in Table 10, the results indicate a significantly negative relationship between PM_2.5_ and component blood donation: when PM_2.5_ increased, the number of blood donations declined. Overall, our model estimation results were relatively reliable.

**TABLE 10:** Alternative modelling.

	(1)	(2)
	**Panel Poisson regression fixed effect**	**Negative binomial**

PM_2.5_	−0.0012***	−0.0014*
	(0.0002)	(0.0007)
Covariates	Y	Y
District FE	Y	Y
Month FE	Y	Y
Dow FE	Y	Y
AIC	8329.35	5849.34
BIC	8445.51	5974.43
N	644	644

The dependent variable is component blood donation. SEs are clustered at the district level. “Y” indicates that these variables are included as predictors in the model. *** and * denote significance at the 0.1 and 5% levels, respectively.

## Discussion

We explored the relationship between air pollution and prosocial behaviour. We merged data on PM_2.5,_ individual blood donations, and weather in the district under study. We used PM_2.5_ to reflect air pollution and used individual blood donation to reflect prosocial behaviour. We employed PQML to estimate the link between air pollution and prosocial behaviour. We also used a series of high-dimensional fixed effects and alternative model specifications to address endogeneity issues and verify robustness.

Our primary finding is that not all prosocial behaviours are significantly associated with air pollution. We found that only component blood donation was significantly associated with air pollution. People aged 30–60 years are more likely to engage in prosocial behaviours, such as component blood donation, and are more likely to be associated with air pollution. In general, our results suggest that there is a significantly negative relationship between air pollution and specific prosocial behaviours (i.e., component blood donation). Furthermore, a significant difference was found in the relationship between air pollution and both blood donation and whole blood donation. Thus, prosocial behaviour is very complex and must be explored in terms of its several categories.

The results indicate that air pollution and prosocial behaviours are not unrelated; they have a complex and close relationship. Only specific prosocial behaviours can be associated with air pollution. Individual blood donation is a good indicator of prosocial behaviour. To examine prosocial behaviour clearly, it is necessary to categorise individual blood donations into whole blood donations and component blood donations. Although both whole and component blood donations can be considered as the types of individual blood donation, such donations imply different body images and cognitive preferences because of the differences in the donation form, donation duration, and interval time. Whole blood donations mainly occur in vans and houses and thus could also be called “street blood donations.” By contrast, component blood donations have a relatively short interval period and occur at fixed blood donation stations. Component blood donations occur in only two districts. As whole and component blood donations have different interval periods, we must count the number of these two blood donations separately. It was thus reasonable to divide individual blood donations into whole and component blood donations in our study. The data we obtained showed that the first blood donation was the main type of whole blood donation and that regular blood donation was the main type of component blood donation. This study found that regular blood donation in component blood donation was significantly associated with PM_2.5_, which could indicate that air pollution may be associated with blood donors who have regular blood donation habits. Our results are largely consistent with those of Zhao et al. (2020), who found that people’s integrity behaviour decreased when air pollution increased. However, we are sceptical of the reliability of their conclusions. First, prosocial behaviour is so complex that it is impossible to identify its association with air pollution based on a few studies alone. In other words, it is necessary to re-examine the association between air pollution and prosocial behaviour. Second, this study mainly used Chinese university students as the research participants, which means the results may not necessarily translate to society as a whole. Third, the quasi-experimental study allowed us to better observe the effects of air pollution on behavioural integrity in the nearest real situation. However, there are still differences from a completely objective and realistic environment. Our conclusion also corroborates the relationship between air pollution and unethical behaviour. A previous study showed that air pollution is negatively associated with violent crimes (Berman et al., 2019). Violent crimes constitute traceable behaviour. Police authorities can find relevant traces of a crime. Our results indicate that air pollution is mainly associated with fixed blood donation in component blood donation. Most regular blood donations are traceable. Blood centres can predict when regular blood donors are likely to donate blood based on their records of past blood donations. From this perspective, it is not surprising to find that air pollution has a negative association with blood donation.

We lacked the data required to identify the mechanism by which air pollution is associated with blood donation. We attempted to explain the reasons for the association between air pollution and blood donations. Component blood donations are mainly given by regular blood donors. Most regular blood donors donate at relatively regular intervals. To be able to donate within the pre-planned time window, they may maintain healthy habits. A connection was observed between the most regular blood donors at the blood centre. They exchanged knowledge and information related to blood donation through WeChat. These donors could be more sensitive to surrounding circumstances and risks. They could cancel or postpone their blood donation plan when PM_2.5_ increases. Regular blood donors may feel strong faith in and responsibility for their blood donation and may also feel that health is the basis of it. Thus, the number of component blood donations may decrease when PM_2.5_ increases. Our results show that most regular blood donors were aged >30 years. We suggest that this is the reason for the difference between donors under 30 and those over 30. Blood donors over 30 may have better physical health management habits than those under 30. In China, most young people aged 18–29 are still students or at the beginning of their careers and are less capable of managing their physical health. Some scholars have found that the youth struggle at this stage of their lives because of many changing pressures. They often lack control over their own lives and require balance. They struggle with health-related issues such as unhealthy eating, lack of sleep, obesity, and lack of exercise. Quick-fix technological solutions are sought to add discipline to their lives, but this is often insufficient in the face of external temptation and low self-management efficacy (Tu and Wang, 2019). Our data show that the majority of first-time blood donors are under 30. The blood donation behaviour of first-time donors may show less of a pattern than that of regular donors. Thus, we did not find a significant association between PM_2.5_ and first-time blood donation. In addition, a previous study found that men expressed less concern for practically all the risks studied. Although men and women seem to worry about the same risks, women worry slightly more (Gustafsod, 1998). From this point of view, it is not surprising that the negative relationship between PM_2.5_ and component blood donation is stronger among women in the same age group (30–60). Finally, we found a moderating role played by daily minimum temperature in the relationship between PM_2.5_ and regular blood donation. Some scholars have found that PM_2.5_ concentration is the highest in winter and the lowest in summer in China (Wang and Fang, 2016; Wang et al., 2017). The lower the temperature, the higher the PM_2.5_ concentration (Xu et al., 2007). There are many reasons for the high PM_2.5_ concentration at lower temperatures, such as incomplete emissions from vehicle exhausts, poor air circulation, and weak pollutant diffusion. Thus, the result indicating that lower daily minimum temperature is linked to a stronger relationship between PM_2.5_ and regular blood donation in component blood donation is reasonable.

This study has several limitations. First, we did not perform a spatiotemporal comparison across years. We used only 1 year of data from H city to study air pollution and prosocial behaviour among various administrative districts. Although H city is a megacity in China and 1 year of data drawn from it can answer many questions, cross-year studies and comparisons with other cities are still necessary. In addition, the mechanism underlying the association between air pollution and specific prosocial behaviours needs to be further explored. Future studies should attempt to use more empirical evidence to examine these mechanisms.

## Data Availability Statement

The raw data supporting the conclusions of this article will be made available by the authors, without undue reservation.

## Author Contributions

SZ: conceptualisation, methodology, software, formal analysis, data curation, writing—original draft preparation, and visualisation. SZ, LW, and ZG: validation, investigation, resources, and writing—review and editing. LW: supervision, project administration, and funding acquisition. ZG: conceptualization, methodology, data curation, writing—original draft preparation, supervision, project administration and funding acquisition of the study. All authors have read and agreed to the published version of the manuscript.

## Conflict of Interest

The authors declare that the research was conducted in the absence of any commercial or financial relationships that could be construed as a potential conflict of interest.

## Publisher’s Note

All claims expressed in this article are solely those of the authors and do not necessarily represent those of their affiliated organizations, or those of the publisher, the editors and the reviewers. Any product that may be evaluated in this article, or claim that may be made by its manufacturer, is not guaranteed or endorsed by the publisher.
